# The effect of growing Rod treatment on coronal balance during serial lengthening surgeries in early onset scoliosis

**DOI:** 10.1186/s12891-016-1007-x

**Published:** 2016-04-12

**Authors:** Wen-jing Li, Zhi-jian Sun, Shi-gong Guo, Gui-xing Qiu, Jian-guo Zhang, Jian-xiong Shen, Yi-peng Wang, Hong Zhao, Shu-gang Li, Yu Zhao

**Affiliations:** Department of Orthopaedics, Peking Union Medical College Hospital, Chinese Academy of Medical Sciences and Peking Union Medical College, Dong Cheng District Shuai Fu Yuan No.1, Beijing, 100730 China; Department of Trauma & Orthopaedic Surgery, Lister Hospital, Stevenage, UK

**Keywords:** Coronal balance, Growing rod, Early onset scoliosis, Lengthening surgery

## Abstract

**Background:**

Gaining and maintaining spinal balance after surgery is of great importance for early onset scoliosis (EOS). However, tendency of balance on the coronal plane after growing rod surgery has not been studied before. This study evaluated the effect of growing rod treatment on coronal balance (CB) during serial lengthening surgeries in EOS.

**Methods:**

All EOS patients treated with growing rod technique in our hospital from August 2002 to June 2014 were retrospectively reviewed. Radiographic data before the sixth lengthening surgery were measured on the posteroanterior X-ray images, including global CB (C7 plumbline-central sacral vertical line, C7PL-CSVL), regional CB (apical vertebrae-CSVL), Cobb angle of the main curve and pelvic inlet width (PIW). Global CB index and regional CB index were calculated as dividing global CB and regional CB by PIW, respectively. The changes of these parameters during repeated lengthening surgeries were analyzed.

**Results:**

Five hundred seventy Radiographs of 67 patients, including 134 images before and after growing rod insertion surgeries and 436 images pre- and post-lengthening surgeries were measured. Global CB and global CB index did not show significant differences between every two set points during lengthening procedures (*P* > 0.05). The percentage of patients with C7PL-CSVL distance more than 20 mm roughly ranged from 30 to 45 % during the lengthening process. With regards to regional CB and main curve Cobb angles, there were significant differences between every two adjacent set points during the first five lengthening surgeries (*P* < 0.05).

**Conclusions:**

Global CB did not significantly change during serial lengthening surgeries and C7PL-CSVL distances of greater than 20 mm comprised of over one third of patients during growing rod treatment. However, worsening regional CB and Cobb angles of the main curve during lengthening intervals were corrected by lengthening manipulation and maintained at a stable level.

## Background

The goal of surgical correction of scoliosis is to achieve a solid spinal fusion in a balanced position in both coronal and sagittal planes. Gaining and maintaining spinal balance after surgery is of great importance as apparent trunk deformity significantly correlates with patients’ quality of life [1–3].

Growing rod surgery was developed to treat early onset scoliosis (EOS) with the goals of obtaining and maintaining deformity correction, achieving adequate spinal growth and allowing sufficient thoracic development [4]. Previous studies had evaluated the effect of growing rod surgery on curve correction, spinal growth as well as thoracic or lung growth [5–19]. Several studies have also evaluated the effect of growing rod treatment on the sagittal profile [20–22]. However, to our knowledge, tendency of trunk balance on the coronal plane during growing rod lengthening surgery has not been studied before.

After growing rod insertion surgery, repeated lengthening surgeries were carried out, which would last for several years. Flynn et al. [23] studied 99 patients who completed lengthening and found that the mean duration of growing rod treatment was 5 years. Coronal profile would influence patients’ quality of life not only after final fusion but also during serial lengthening surgeries. During serial lengthening procedures, long term coronal imbalance would probably influence EOS patients’ quality of life. Therefore we retrospectively reviewed the radiographic data of EOS patients treated with growing rod in our hospital and conducted this study to evaluate the effect of consecutive growing rod lengthening on coronal balance (CB).

## Methods

### Patients

All EOS patients treated with growing rods in our hospital from August 2002 to June 2014 were retrospectively reviewed. Patients who underwent at least one lengthening surgery were included. Patients with kyphoscoliosis were excluded. Since very few patients underwent more than five lengthenings, radiographic data up to and including the fifth lengthening surgery were reviewed.

If the radiographic parameters could not be measured due to technical inadequacy of the plain film, measurements and outcomes for that specific radiograph were excluded from our study. In addition, if patients underwent final fusion surgeries before the fifth lengthening surgery, radiographs after the final surgery were not measured. This study was approved by the institutional review board of Peking Union Medical College Hospital. And written informed consent was obtained from all participants in the study.

### Radiographic measurements

**Fig. 1:**
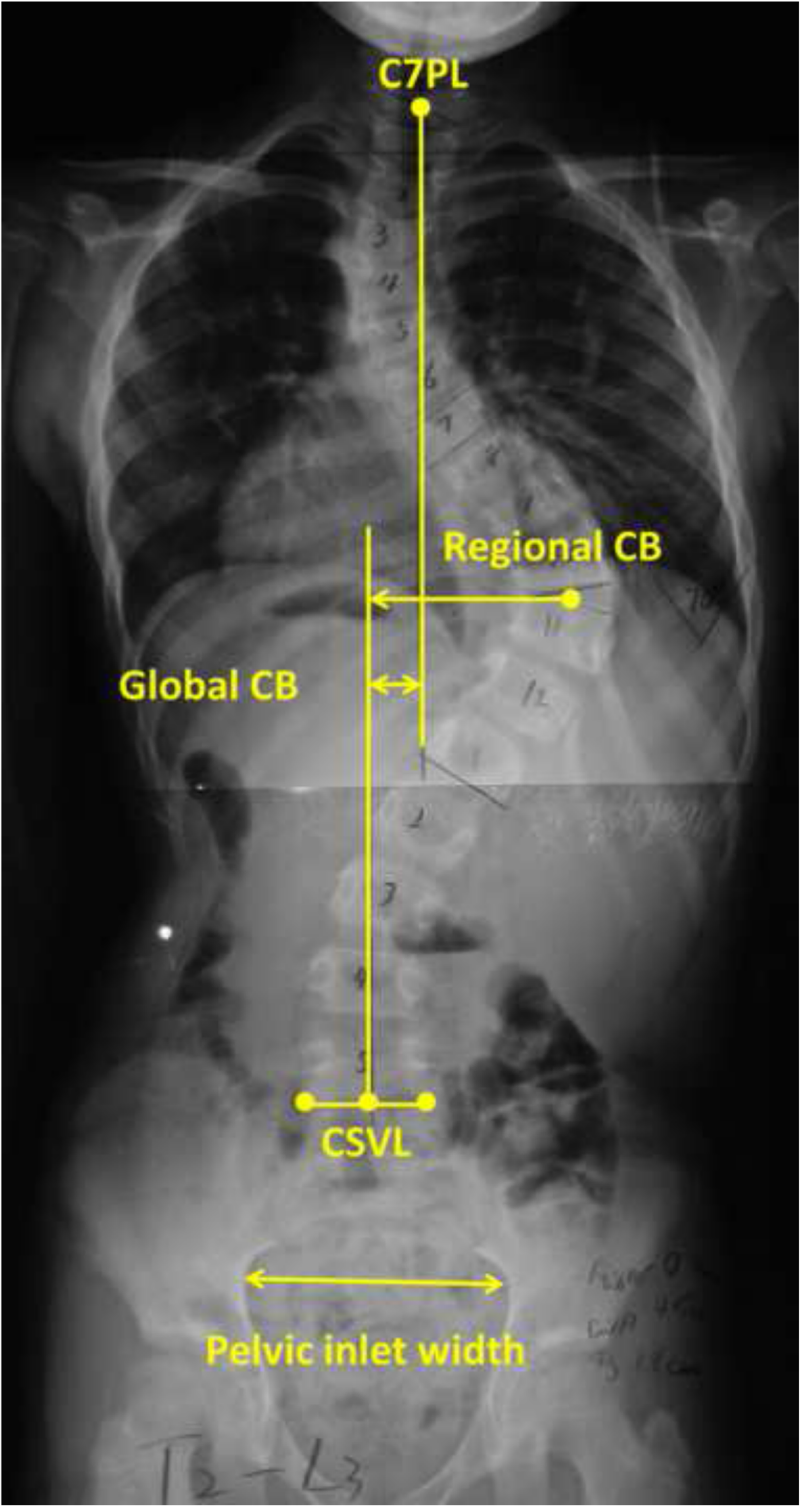
Measurements of global coronal balance, regional coronal balance and pelvic inlet width

The upright posteroanterior images before and after growing rod insertion surgery and the first five lengthening surgeries were measured, including global CB, regional CB, Cobb angle of the main curve and pelvic inlet width (PIW). Global CB was measured in millimeters as the perpendicular distance between the C7 plumbline and the central sacral vertical line (C7PL–CSVL). Regional CB was defined as the perpendicular distance between the midpoint of the apex vertebrae of the main curve and CSVL (AV-CSVL). PIW was measured at the maximal width across the inner margin of the left and right iliac bones [24] (Fig. 1). PIW is an age-independent parameter, which can be used as an individualized internal standard of growth [25]. Thus, global CB index and regional CB index were calculated as dividing global CB and dividing regional CB by PIW, respectively.

Measurements were performed by two of authors independently using Surgimap version 1.2.1.86 (Nemaris Inc, New York, NY, USA). All the radiographs were calibrated to achieve accurate distance measurements. The alignments of all stitched films were carefully checked.

### Statistical analysis

Statistical analysis was conducted using SAS software version 9.4 (SAS Institute, Inc., Cary, NC). Comparisons of global CB, regional CB, Cobb angle of the main curve, global CB index and regional CB index at adjacent set points were performed using covariance analysis. *P* values of less than 0.05 were considered statistically significant.

## Results

**Fig. 2:**
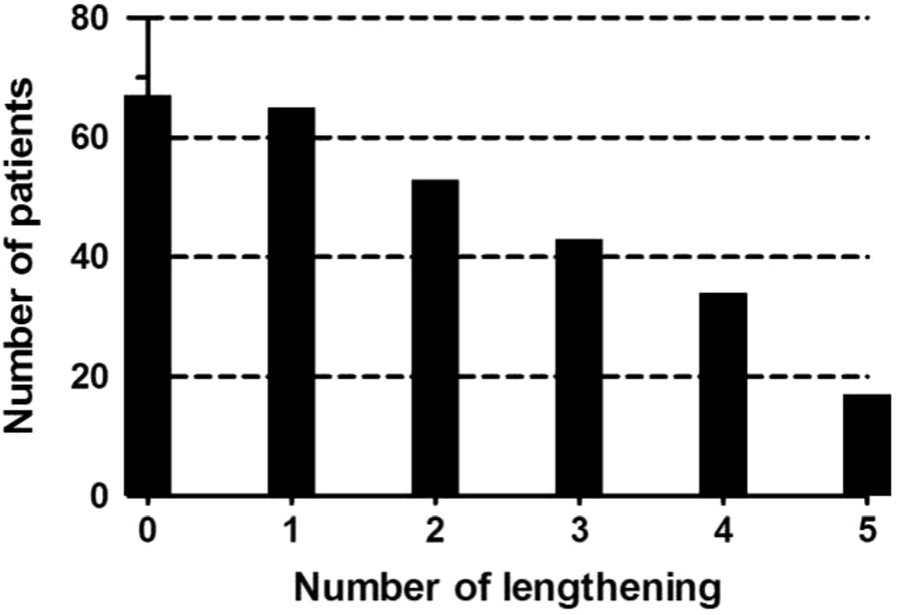
Numbers of patients that underwent initial growing rod insertion and each subsequent lengthening “0” indicates growing rod insertion surgery and “1-5” indicate the first to the fifth lengthening surgery respectively

A total of 67 patients, including 21 males and 46 females were included in this study. The mean age at initial growing rod insertion surgeries was 7.3 ± 2.9 years (range from 2 to 13 years). And the mean duration of growing rod treatment was 2.8 ± 1.5 years (range from 0.5 to 6 years). An average of 3.4 lengthening procedures per patient with an average interval of 0.8 years was performed. Of the 67 patients, 59 underwent dual growing rod surgery and 8 received single growing rod treatment. In terms of etiology, 52 patients were congenital (77.6 %), 4 patients were idiopathic (5.9 %), 3 patients had an underlying neuromuscular condition (4.4 %), 5 patients had neurofibromatosis(7.5 %) and 3 patients had syndromic scoliosis (4.4 %). 570 Radiographs were available for measurement, including 134 images before and after growing rod insertion surgeries and 436 images pre- and post-lengthening surgeries. The number of patients that underwent initial growing rod insertion and each subsequent lengthening is illustrated in Fig. 2.

**Fig. 3:**
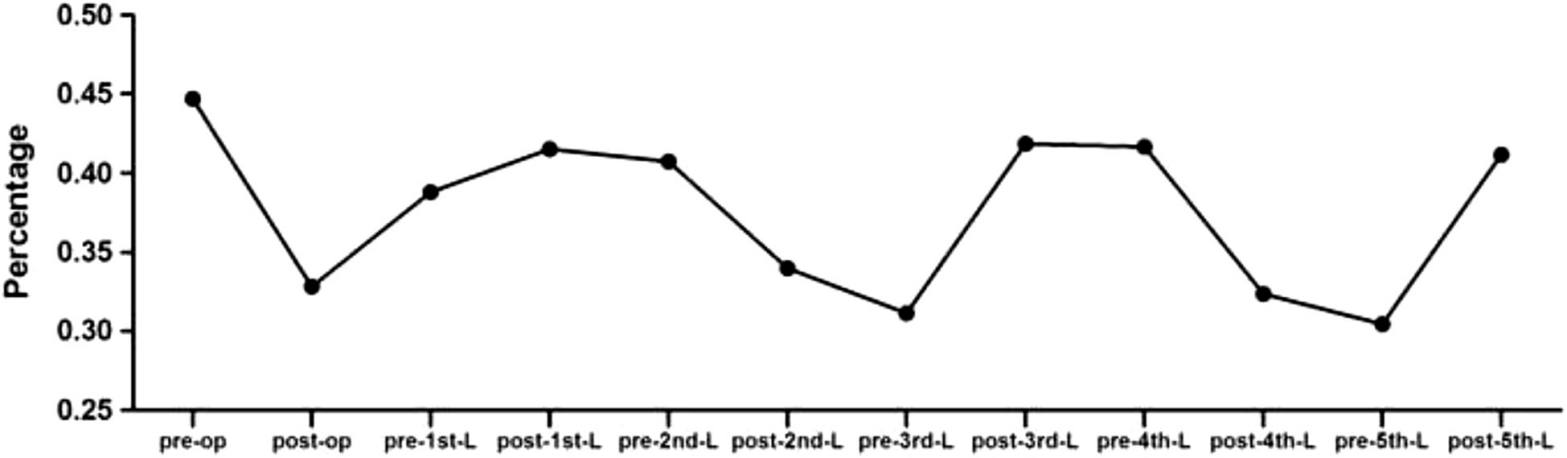
Percentage of patients with C7PL-CSVL distance greater than 20 mm during growing rod treatment

**Fig. 4:**
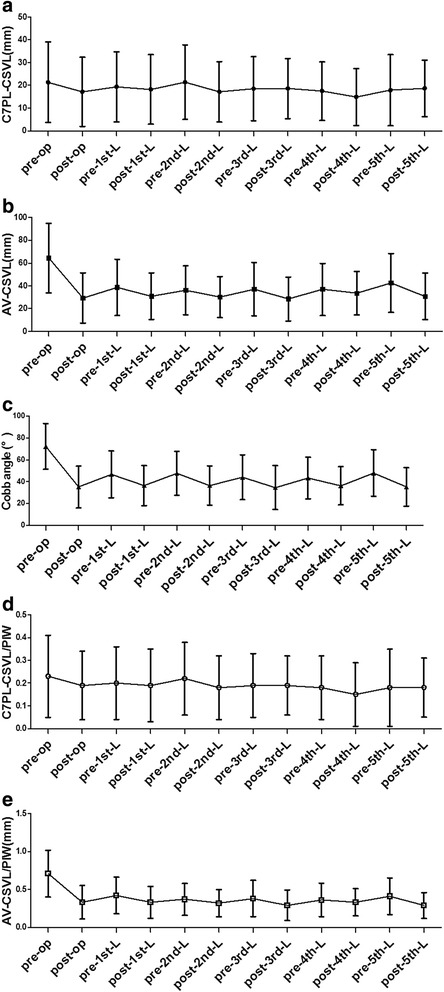
Changes in CB of initial surgeries and consecutive lengthening surgeries in EOS patients

The mean preoperative global CB of the 67 patients was 21.3 ± 17.7 mm, and C7PL-CSVL distances were greater than 20 mm in 30 patients (44.7 %) (Fig. 3) After growing rod insertion surgery, the mean global CB changed to 17.1 ± 15.2 mm, which showed significant differences (*P* = 0.0382, Table 1). The percentage of C7PL-CSVL distance greater than 20 mm also fell to 32.8 % (22/67). However, during lengthening procedures, global CB did not show significant differences between every two set points (*P* > 0.05; Table 1, Fig. 4a). The percentage of patients with C7PL-CSVL distance >20 mm also showed no regularity, approximately ranging from 30 to 45 % (Fig. 3). When normalized by PIW, global CB index did not show statistical difference both between measurements of initial surgery and between lengthening surgeries (*P* > 0.05; Table 1, Fig. 4d).

**Table 1:** Comparison of CB during serial growing rod lengthening procedures in EOS patients

Comparison	*P* value				
	Global CB	Regional CB	Cobb angle	Global CB index	Regional CB index
Pre-op vs. post-op	**0.0382**	**<.0001**	**<.0001**	**0.0361**	**<.0001**
Post-op vs. pre-1^st^-L	0.2732	**<.0001**	**<.0001**	0.5564	**<.0001**
Pre-1^st^-L vs. post-1^st^-L	0.5651	**0.0004**	**<.0001**	0.5237	**<.0001**
Post-1^st^-L vs. pre-2^nd^-L	0.1694	**0.0268**	**<.0001**	0.1653	0.1291
Pre-2^nd^-L vs. post-2^nd^-L	0.0762	**0.0080**	**<.0001**	0.0579	**0.0119**
Post-2^nd^-L vs. pre-3^rd^-L	0.2604	**0.0025**	**<.0001**	0.4004	**0.0162**
Pre-3^rd^-L vs. post-3^rd^-L	0.8247	**0.0006**	**<.0001**	0.9603	**0.0005**
Post-3^rd^-L vs. pre-4^th^-L	0.7842	**0.0101**	**<.0001**	0.7810	0.0886
Pre-4^th^-L vs. post-4^th^-L	0.3001	0.1113	**0.0003**	0.2641	0.1171
Post-4^th^-L vs. pre-5^th^-L	0.5719	**0.0134**	**0.0002**	0.6989	0.0565
Pre-5^th^-L vs. post-5^th^-L	0.3782	**0.0362**	**0.0008**	0.3825	0.0679

Bold numbers were considered statistically significant (*P* > 0.05)

With regards to regional CB, there were significant differences between every two adjacent set points of initial surgeries and the first five lengthening surgeries, which was more obvious before and after initial surgeries with the mean AV-CSVL distance changing from 64.5 ± 30.5 mm to 29.4 ± 22.2 mm (*P* < 0.05; Table 1, Fig. 4b). However, they fluctuated at a certain range during the following lengthening surgeries, which illustrated as a wavy curve in Fig. 4b. Regional CB index also showed significant differences before and after initial growing rod insertion surgeries, after initial surgeries and before first lengthening as well as before and after first lengthening (*P* < 0.05; Table 1, Fig. 4e). Although the wavy curve changes still existed during subsequent lengthening surgeries, no statistical difference was observed (*P* > 0.05; Table 1, Fig. 4e).

The mean preoperative Cobb angle of the main curve was 72.3° ± 20.9°, ranging from 32°to 135°, which was corrected to an average of 37.2° ± 13.6° after initial surgeries (*P* < 0.05). Then a wavy change was also observed during the following lengthening surgeries, which manifested an increase during lengthening intervals and a decrease after lengthening manipulations (Table 1, Fig. 4c).

Twenty implant-related complications occurred in 12 out of 67 patients, including 7 hook dislodgements, 7 screw pullouts and 6 rod breakages. All these complications were corrected during subsequent planned elective lengthening procedures. No patients experienced wound infection or other medical complications.

## Discussion

Other than deformity correction and maintaining spinal and thoracic growth, CB of EOS patients is also an important aspect during growing rod treatment. Normally this problem can be easily ignored, considering CB during lengthening surgeries is temporary and does not represent the final result. However, the whole growing rod treatment usually lasts for many years [6, 23]. Coronal imbalance during this long period would probably influence EOS patients’ quality of life [26]. In this study, through analyzing the radiographic data of 67 EOS patients treated by growing rod technique, we found that global CB was maintained during serial lengthening procedures whereas worsened regional CB during lengthening intervals was corrected by lengthening manipulations.

Global CB, defined as C7PL-CSVL distance, was affected by several factors, including compensative changes of uninvolved segments cephalad to the upper instrumented vertebrae and caudad to the lower instrumented vertebrae, aggravating tendencies of the main curve due to the Hueter-Volkmann law and inherent etiologies, restriction of involved segments by growing rod constructs, and regular lengthening manipulations [27]. Although C7PL-CSVL distance significantly decreased immediately after growing rod insertion surgery,the overall effect of these aforementioned factors ensured that global CB was generally stable throughout serial lengthening procedures in our study.

Normally, global coronal imbalance is defined as C7PL-CSVL distance greater than 20 mm in adolescent idiopathic scoliosis [3, 28, 29]. Using this definition, we might underestimate coronal imbalance in EOS patients considering their smaller size than in adolescents. Nevertheless, the percentage of C7PL-CSVL distance greater than 20 mm was also analyzed. It turned out comparatively, there were a greater proportion of patients with C7PL-CSVL distance more than 20 mm during growing rod treatment, and the effect of growing rod manipulation on global CB during repeated lengthenings was very limited. However, we speculated that global CB would probably improve after final fusion surgery [30].

Regional CB, defined by AV-CSVL distance in this study, was also affected by similar factors as global CB, except compensative changes of uninvolved segments that were cephalad to the upper instrumented vertebrae. We hypothesized that worsening of the main curve during lengthening intervals might play a vital role, because in this study we found that regional CB was aggravated during lengthening intervals and growing rod lengthening manipulation significantly reversed it. However, regional CB index did not show statistical difference between every two set point measurements after the first lengthening surgeries. Thus, spinal growth might also have some effects on regional CB, which should be studied further in the future.

As expected, Cobb angle of the main curve was increased during lengthening intervals because of aggravating tendencies of the curve itself and restriction of growing rod constructs. Lengthening manipulations reversed the deterioration and maintained it in a stable level.

There were some limitations in this study. First, the enrolled patients have different etiologies with varying magnitude of the main curve Cobb angle (ranging from 32° to 135°), which might influence the statistical results of change tendencies of global CB and regional CB during consecutive lengthening procedures. In addition, only the first five lengthening radiographic data was analyzed due to the small number of patients that received more than five lengthening surgeries [19]. However, we could still analyze the effect of growing rod treatment on CB during subsequent lengthenings based on the findings of this study. Finally, complications, especially implant-related complications, could also affect global and regional CB. We did not exclude these patients or the specific surgeries, thus the results of this study reflected the overall effect of growing rod treatment on CB, and not just lengthening manipulations.

## Conclusions

In conclusion, through analyzing 570 radiographic data of 67 EOS patients treated with growing rod technique, we found that global CB did not significantly change during serial lengthening surgeries and approximately 30–45 % patients had C7PL-CSVL distance greater than 20 mm. Regional CB and Cobb angle of the main curve worsened during lengthening intervals, both of which were corrected by lengthening manipulation and thereby maintained at a stable level.

## Ethics approval and consent to participate

This study was approved by the institutional review board of Peking Union Medical College Hospital. And written informed consent was obtained from all participants in the study.

## Consent for publication

Not applicable.

## Availability of data and materials

The dataset supporting the conclusions of this article is available on request to the corresponding author.

## Abbreviations

AV-CSVL: Apical vertebrae-central sacral vertical line
C7PL-CSVL: C7 plumbline-central sacral vertical line
CB: Coronal balance
EOS: Early onset scoliosis
PIW: Pelvic inlet width

## References

[1] Asher M, Lai SM, Burton D, Manna B (2004). The influence of spine and trunk deformity on preoperative idiopathic scoliosis patients' health-related quality of life questionnaire responses. Spine (Phila Pa 1976).

[2] Glassman SD, Berven S, Bridwell K, Horton W, Dimar JR (2005). Correlation of radiographic parameters and clinical symptoms in adult scoliosis. Spine (Phila Pa 1976).

[3] Trobisch PD, Samdani AF, Pahys JM, Cahill PJ (2011). Postoperative trunk shift in Lenke 1 and 2 curves: how common is it? and analysis of risk factors. Eur Spine J.

[4] Tis JE, Karlin LI, Akbarnia BA, Blakemore LC, Thompson GH, McCarthy RE (2012). Early onset scoliosis: modern treatment and results. J Pediatr Orthop.

[5] Akbarnia BA, Marks DS, Boachie-Adjei O, Thompson AG, Asher MA (2005). Dual growing rod technique for the treatment of progressive early-onset scoliosis: a multicenter study. Spine (Phila Pa 1976).

[6] Akbarnia BA, Breakwell LM, Marks DS, McCarthy RE, Thompson AG, Canale SK (2008). Dual growing rod technique followed for three to eleven years until final fusion: the effect of frequency of lengthening. Spine (Phila Pa 1976).

[7] Sponseller PD, Yang JS, Thompson GH, McCarthy RE, Emans JB, Skaggs DL (2009). Pelvic fixation of growing rods: comparison of constructs. Spine (Phila Pa 1976).

[8] Sabourin M, Jolivet E, Miladi L, Wicart P, Rampal V, Skalli W (2010). Three-dimensional stereoradiographic modeling of rib cage before and after spinal growing rod procedures in early-onset scoliosis. Clin Biomech (Bristol, Avon).

[9] McElroy MJ, Shaner AC, Crawford TO, Thompson GH, Kadakia RV, Akbarnia BA (2011). Growing rods for scoliosis in spinal muscular atrophy: structural effects, complications, and hospital stays. Spine (Phila Pa 1976).

[10] Jiang Y, Zhao Y, Wang YP, Qiu GX, Weng XS, Li Y (2011). Lung function after growing rod surgery for progressive early-onset scoliosis: a preliminary study. Chin Med J (Engl).

[11] Elsebai HB, Yazici M, Thompson GH, Emans JB, Skaggs DL, Crawford AH (2011). Safety and efficacy of growing rod technique for pediatric congenital spinal deformities. J Pediatr Orthop.

[12] Wang S, Zhang J, Qiu G, Wang Y, Li S, Zhao Y (2012). Dual growing rods technique for congenital scoliosis: more than 2 years outcomes: preliminary results of a single center. Spine (Phila Pa 1976).

[13] McElroy MJ, Sponseller PD, Dattilo JR, Thompson GH, Akbarnia BA, Shah SA (2012). Growing rods for the treatment of scoliosis in children with cerebral palsy: a critical assessment. Spine (Phila Pa 1976).

[14] Caniklioglu M, Gokce A, Ozturkmen Y, Gokay NS, Atici Y, Uzumcugil O (2012). Clinical and radiological outcome of the growing rod technique in the management of scoliosis in young children. Acta Orthop Traumatol Turc.

[15] Zhao Y, Qiu GX, Wang YP, Zhang JG, Shen JX, Li SG (2012). Comparison of initial efficacy between single and dual growing rods in treatment of early onset scoliosis. Chin Med J (Engl).

[16] Karatas AF, Dede O, Rogers K, Ditro CP, Holmes L, Bober M (2013). Growth-sparing spinal instrumentation in skeletal dysplasia. Spine (Phila Pa 1976).

[17] Kamaci S, Demirkiran G, Ismayilov V, Olgun ZD, Yazici M (2014). The effect of dual growing Rod instrumentation on the apical vertebral rotation in early-onset idiopathic scoliosis. J Pediatr Orthop.

[18] Wang S, Zhang J, Qiu G, Wang Y, Weng X, Guo J (2014). One-stage posterior osteotomy with short segmental fusion and dual growing rod technique for severe rigid congenital scoliosis: the preliminary clinical outcomes of a hybrid technique. Spine (Phila Pa 1976).

[19] Sun ZJ, Qiu GX, Zhao Y, Guo SG, Zhang JG, Shen JX (2015). Dual growing rod treatment in early onset scoliosis: the effect of repeated lengthening surgeries on thoracic growth and dimensions. Eur Spine J.

[20] Shah SA, Karatas AF, Dhawale AA, Dede O, Mundis GJ, Holmes LJ (2014). The effect of serial growing rod lengthening on the sagittal profile and pelvic parameters in early-onset scoliosis. Spine (Phila Pa 1976).

[21] Watanabe K, Uno K, Suzuki T, Kawakami N, Tsuji T, Yanagida H, et al. Risk factors for proximal junctional Kyphosis associated with dual-Rod growing-Rod surgery for early-onset scoliosis. J Spinal Disord Tech. 2014. 10.1097/BSD.0000000000000127 25023715

[22] Atici Y, Akman YE, Erdogan S, Sari S, Yavuz U, Carkci E (2015). The effect of growing rod lengthening technique on the sagittal spinal and the spinopelvic parameters. Eur Spine J.

[23] Flynn JM, Tomlinson LA, Pawelek J, Thompson GH, McCarthy R, Akbarnia BA (2013). Growing-rod graduates: lessons learned from ninety-nine patients who completed lengthening. J Bone Joint Surg Am.

[24] Gold M, Dombek M, Miller PE, Emans JB, Glotzbecker MP (2014). Prediction of thoracic dimensions and spine length on the basis of individual pelvic dimensions: validation of the use of pelvic inlet width obtained by radiographs compared with computed tomography. Spine (Phila Pa 1976).

[25] Emans JB, Ciarlo M, Callahan M, Zurakowski D (2005). Prediction of thoracic dimensions and spine length based on individual pelvic dimensions in children and adolescents: an age-independent, individualized standard for evaluation of outcome in early onset spinal deformity. Spine (Phila Pa 1976).

[26] Flynn JM, Matsumoto H, Torres F, Ramirez N, Vitale MG (2012). Psychological dysfunction in children who require repetitive surgery for early onset scoliosis. J Pediatr Orthop.

[27] Olgun ZD, Ahmadiadli H, Alanay A, Yazici M (2012). Vertebral body growth during growing rod instrumentation: growth preservation or stimulation?. J Pediatr Orthop.

[28] Sun Z, Qiu G, Zhao Y, Wang Y, Zhang J, Shen J (2014). Lowest instrumented vertebrae selection for selective posterior fusion of moderate thoracolumbar/lumbar idiopathic scoliosis: lower-end vertebra or lower-end vertebra+1?. Eur Spine J.

[29] Demura S, Yaszay B, Bastrom TP, Carreau J, Newton PO (2013). Is decompensation preoperatively a risk in Lenke 1C curves?. Spine (Phila Pa 1976).

[30] Sun Z, Qiu G, Zhao Y, Guo S, Wang Y, Zhang J (2014). The effect of unfused segments in coronal balance reconstitution after posterior selective thoracolumbar/lumbar fusion in adolescent idiopathic scoliosis. Spine (Phila Pa 1976).

